# Development of Thermosensitive Hydrogels of Chitosan, Sodium and Magnesium Glycerophosphate for Bone Regeneration Applications

**DOI:** 10.3390/jfb6020192

**Published:** 2015-04-09

**Authors:** Jana Lisková, Lucie Bačaková, Agata L. Skwarczyńska, Olga Musial, Vitaliy Bliznuk, Karel De Schamphelaere, Zofia Modrzejewska, Timothy E.L. Douglas

**Affiliations:** 1 Department of Biomaterials and Tissue Engineering, Institute of Physiology of the Czech Academy of Sciences, Videnska 1083, Prague, Czech Republic; E-Mail: janefox@biolog.cz (J.L.); lucy@biomed.cas.cz (L.B.); 2 Faculty of Civil Environmental Engineering and Architecture, Rzeszow University of Technology, ul. Poznanska 2, Rzeszow 35-084, Poland; E-Mail: askwarczynska1@wp.pl; 3 Polymer Chemistry and Biomaterials (PBM) Group, Department of Organic Chemistry, Ghent University, Krijgslaan 281 S4, Gent 9000, Belgium; E-Mail: olga.musial@op.pl; 4 Department Material Science & Engineering, Ghent University, Technologiepark 903, Zwijnaarde 9052, Belgium; E-Mail: vitaliy.bliznuk@ugent.be; 5 Laboratory for Environmental and Aquatic Ecology, Environmental Toxicology Unit (GhEnToxLab), Faculty of Bioscience Engineering, Ghent University, Jozef Plateaustraat 22, Gent 9000, Belgium; E-Mail: Karel.Deschamphelaere@ugent.be; 6 Department of Environmental Systems Engineering, Faculty of Process and Environmental Engineering, Technical University of Łódź, ul. Wólczańska 213, Łódź 90-924, Poland; E-Mail: zofia.modrzejewska@p.lodz.pl

**Keywords:** chitosan, hydrogel, cytocompatibility, magnesium, mineralization

## Abstract

Thermosensitive injectable hydrogels based on chitosan neutralized with sodium beta-glycerophosphate (Na-β-GP) have been studied as biomaterials for drug delivery and tissue regeneration. Magnesium (Mg) has been reported to stimulate adhesion and proliferation of bone forming cells. With the aim of improving the suitability of the aforementioned chitosan hydrogels as materials for bone regeneration, Mg was incorporated by partial substitution of Na-β-GP with magnesium glycerophosphate (Mg-GP). Chitosan/Na-β-GP and chitosan/Na-β-GP/Mg-GP hydrogels were also loaded with the enzyme alkaline phosphatase (ALP) which induces hydrogel mineralization. Hydrogels were characterized physicochemically with respect to mineralizability and gelation kinetics, and biologically with respect to cytocompatibility and cell adhesion. Substitution of Na-β-GP with Mg-GP did not negatively influence mineralizability. Cell biological testing showed that both chitosan/Na-β-GP and chitosan/Na-β-GP/Mg-GP hydrogels were cytocompatible towards MG63 osteoblast-like cells. Hence, chitosan/Na-β-GP/Mg-GP hydrogels can be used as an alternative to chitosan/Na-β-GP hydrogels for bone regeneration applications. However the incorporation of Mg in the hydrogels during hydrogel formation did not bring any appreciable physicochemical or biological benefit.

## 1. Introduction

Thermosensitive injectable chitosan hydrogels can be formed by mixing acidic chitosan solutions with the weak base sodium beta-glycerophosphate (Na-β-GP) at low temperature (4 °C) followed by raising the temperature to body temperature [1,2]. Neutralization of the acid with Na-β-GP reduces electrostatic repulsion between the positively charged chitosan molecules. The raise in temperature leads to breaking of intramolecular hydrogen bonds, resulting in a more “stretched out” configuration of the chitosan molecules. Na-β-GP enhances dehydration of chitosan molecules and helps to remove H^+^ ions from chitosan molecules, which facilitate hydrophobic interactions and hydrogen bonding between chitosan molecules [3,4]. All the aforementioned effects facilitate formation of a chitosan hydrogel.

The presence of a calcium phosphate (CaP) component in hydrogels is considered desirable to improve their suitability as materials for bone regeneration [5]. Indeed, the presence of apatite in hydrogels has improved proliferation [6] and osteogenic differentiation [7,8] of mesenchymal stem cells (MSC) *in vitro* and bone formation *in vivo*, both in rat cranial critical defects [7,9] and ectopically [10].

In a previous study, chitosan/Na-β-GP hydrogels were enriched with the enzyme alkaline phosphatase (ALP), which led to acceleration of gelation and promotion of formation of CaP within the hydrogel [11]. CaP formed as a result of incorporation of ALP into the hydrogel polymer network during hydrogel formation followed by incubation in a solution containing calcium ions and glycerophosphate, a substrate for ALP. This approach has been applied to mineralize a range of other hydrogels [12,13,14,15,16] and can be considered to be bioinspired, as mineralization of bone tissue with CaP *in vivo* is caused by ALP.

In this study, it was hypothesized that the enrichment of chitosan hydrogels with magnesium would enhance adhesion and proliferation of osteoblast-like cells seeded on the hydrogels. Addition of magnesium to hydroxyapatite (HA) materials has stimulated osteoblast adhesion [17] and proliferation [18,19,20] *in vitro*. In previous work, incorporation of magnesium into mineral formed in gellan gum hydrogels led to enhanced osteoblast-like cell adhesion and proliferation [15]. An *in vivo* study showed that magnesium enrichment of HA coatings on titanium implants led to improved early osseointegration [21].

To incorporate magnesium into chitosan hydrogels, neutralization was performed not only with Na-β-GP, but with a combination of Na-β-GP and magnesium glycerophosphate (Mg-GP). Hydrogels were characterized physicochemically with respect to gelation speed, mineralizability in a simulated body fluid (SBF) and type of mineral formed. Cell biological characterization was conducted using osteoblastic-like cells.

## 2. Results and Discussion

### 2.1. Physiochemical Characterization

**Figure 1:**
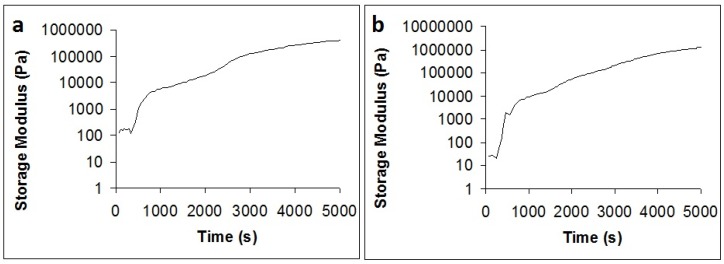
Gelation kinetics of chitosan/sodium beta-glycerophosphate (Na-β-GP) (**a**) and chitosan/Na-β-GP/magnesium glycerophosphate (Mg-GP) (**b**) hydrogels containing 2.5 mg/mL alkaline phosphatase (ALP).

Rheometry results (Figure 1) showed that the gelation speeds of both hydrogel types were comparable. Since the concentration of GP in both hydrogel types was approximately equal, this result means that the partial replacement of Na^+^ ions with Mg^2+^ ions does not influence gelation. 

**Figure 2:**
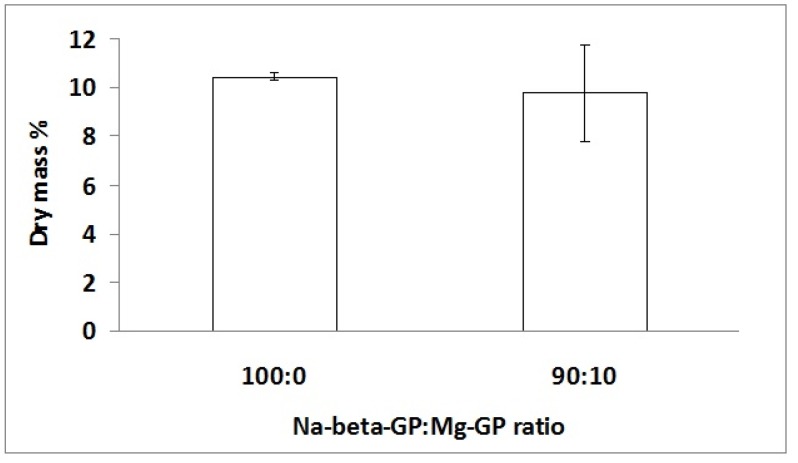
Dry mass percentage of chitosan/Na-β-GP (100:0) and chitosan/Na-β-GP/Mg-GP (90:10) hydrogels containing 2.5 mg/mL ALP after gelation for 1 day followed by incubation for 14 days in a simulated body fluid (SBF). Mean values are shown (*n* = 3). Error bars show standard deviation.

Dry mass percentage measurements (Figure 2) revealed no significant difference in amount of mineral formed in the two hydrogel types. AAS measurements revealed that the amounts of elemental Ca present in freeze-dried hydrogels were 37 and 24 μg/mg sample for chitosan/Na-β-GP and chitosan/Na-β-GP/Mg-GP hydrogels, respectively.

**Figure 3:**
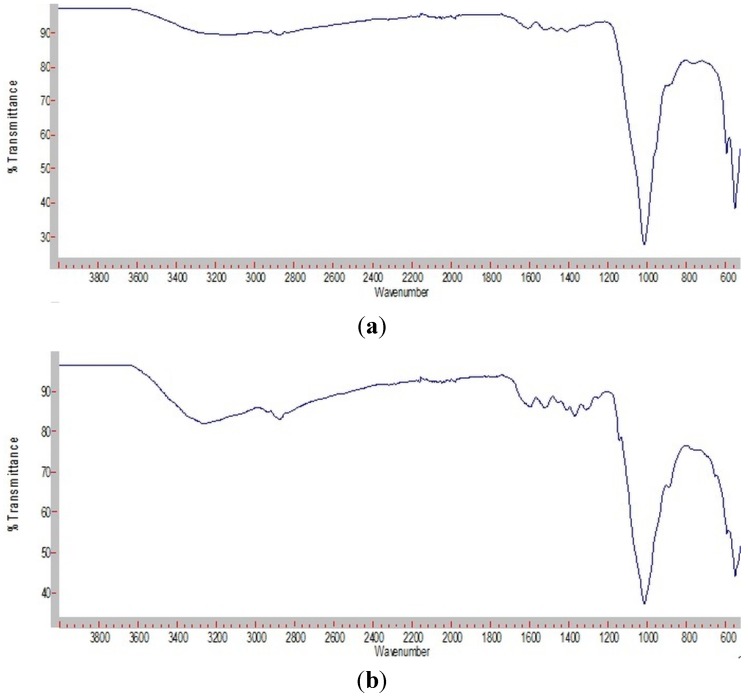
FTIR spectra of lyophilized of chitosan/Na-β-GP (**a**) and chitosan/Na-β-GP/Mg-GP (**b**) hydrogels containing 2.5 mg/mL ALP after incubation for 14 days in a simulated body fluid (SBF).

ATR-FTIR analysis (Figure 3) demonstrated that the mineral formed in both hydrogel types was apatite. Bands characteristic for phosphate υ^4^ and υ^3^ stretching were seen in the regions 600–500 cm^−1^ and 1200–800 cm^−1^, respectively [22]. Both hydrogel types showed a sharp and very intensive band at approximately 1020 cm^−1^ and a double band at approximately 540 and 600 cm^−1^. These are typical for apatite [22,23].

**Figure 4:**
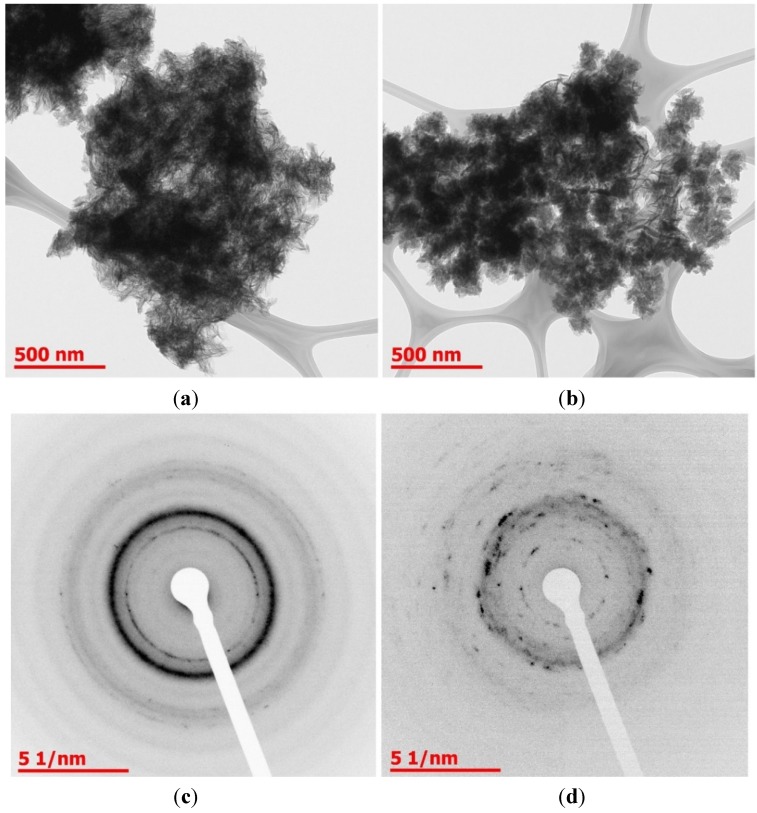
TEM images of mineral deposits in lyophilized chitosan/Na-β-GP (**a**) and chitosan/Na-β-GP/Mg-GP (**b**) hydrogels containing 2.5 mg/mL ALP after incubation for 14 days in a simulated body fluid (SBF). SAED patterns of mineral deposits in lyophilized chitosan/Na-β-GP (**c**) and chitosan/Na-β-GP/Mg-GP (**d**) hydrogels.

TEM and SAED analysis (Figure 4) also demonstrated the presence of a crystalline phase. In chitosan/Na-β-GP hydrogels, needle-like crystals characteristic of calcium-deficient apatite were seen [24]. Diffraction rings corresponding to the (002) and (211) planes of apatite were observed [25,26]. In chitosan/Na-β-GP/Mg-GP hydrogels, needle-like crystals were also seen, however diffraction rings in the SAED pattern were less sharp, suggesting that the deposit analyzed may have been less crystalline.

It is known that magnesium inhibits apatite formation and stabilizes amorphous calcium phosphate both in solution [22,27,28,29] and in hydrogels [15]. The fact that apatite was formed in both hydrogel types, and the fact that amount of mineral formed was similar in both hydrogel types, both suggest that the magnesium added to the chitosan/Na-β-GP/Mg-GP hydrogels was insufficient to hinder apatite formation.

### 2.2. Cell Biological Characterization

**Figure 5:**
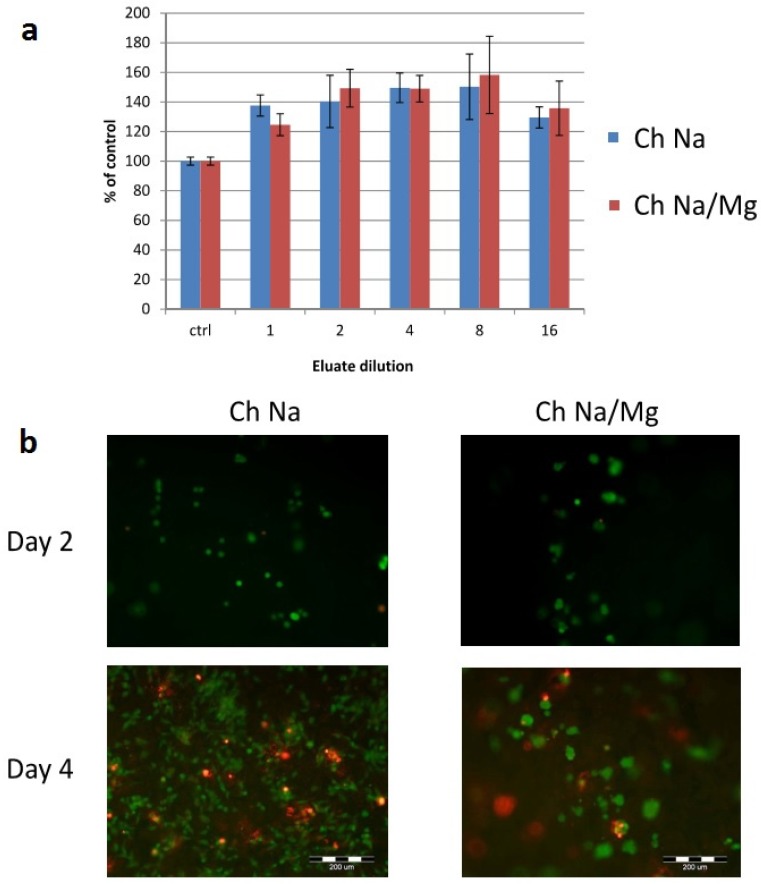
Cytocompatibility and adhesion of MG63 cells. (**a**) Mitochondrial activity of cells cultured in dilutions of eluates of chitosan/Na-β-GP (Ch Na) and chitosan/Na-β-GP/Mg-GP (Ch Na/Mg) hydrogels. Mean values (*n* = 3) error bars show standard deviation; (**b**) Live-Dead staining of cells cultured directly on hydrogels after 2 and 4 days.

Experiments with osteoblast-like cells (Figure 5) revealed that neither hydrogel type exhibited cytotoxicity when eluated in the medium. The eluates even slightly supported cell growth (Figure 5a). After 2 days, viable cells were observed on both hydrogel types (Figure 5b). After 4 days, both viable and dead cells were observed on both hydrogel types. In another study, human adipose-derived stem cells were encapsulated in chitosan/Na-β-GP hydrogels of similar composition to those used in this study [30]. After 7 days, both live and dead cells were observed.

Both hydrogel types showed good and comparable cytocompatibility (Figure 5). Kim *et al.* seeded rat muscle-derived stem cells onto chitosan hydrogels neutralized with Na-β-GP [31]. Final concentrations of chitosan and Na-β-GP were 2% and 20% w/v, respectively. Chitosan hydrogels supported adhesion and proliferation of cells from 1 to 21 days of culture.

The addition of magnesium to scaffolds did not appear to lead to improved adhesion or cell number. It is possible that the amount of magnesium added was too low to influence cell adhesion and proliferation. The content of Mg-GP in the initial chitosan/Na-β-GP/Mg-GP hydrogels was 0.9% w/w, which corresponds to an elemental magnesium content of 0.11% w/w. For comparison, the magnesium contents in bone, dentine and enamel are 0.47%, 1.11% and 0.3% (all w/w), respectively [32]). Several groups have reported a positive effect of magnesium on osteoblast adhesion and proliferation on biomaterials [17,19,33,34,35,36]. In these studies, the biomaterials contained similar amounts of magnesium as the chitosan/Na-β-GP/Mg-GP hydrogels used in this study. However, these biomaterials were solid ceramic materials and not hydrogels. It is also possible that magnesium may have leached out of the hydrogel during cell culture.

## 3. Experimental Section 

### 3.1. Hydrogel Production

Unless stated otherwise, all chemicals were obtained from Sigma-Aldrich, including chitosan powder [shrimp-derived, deacetylation degree (DD) 83.4%, molecular weight (Mw) 862 kDa], HCl, glycerol, ALP (bovine intestinal mucosa-derived product no. P7640), Na-β-GP (product number 50020) and Mg-GP (product number 17766).

Chitosan was dissolved in 0.1 M HCl at a concentration of 25 mg/mL. 1 g Na-β-GP was mixed with 1 mL of MilliQ-water to yield a 1 g/mL Na-β-GP solution-suspension. 0.9 g Na-β-GP and 0.09 g Mg-GP were mixed with 1 mL of MilliQ-water to yield a Na-β-GP/Mg-GP solution-suspension. The concentration of GP in both solution-suspensions was approximately equal. Chitosan solutions and Na-β-GP and Na-β-GP/Mg-GP solution-suspensions were mixed for 24 h under rotation. ALP was dissolved in MilliQ-water at a concentration of 25 mg/mL. 4 mL chitosan solution, 0.4 mL Na-β-GP or Na-β-GP/Mg-GP solution-suspension, and 0.4 mL ALP solution were mixed together using a pipette to yield 4.4 mL hydrogels. Gelation took place in tubes of inner diameter 8 mm at 37 °C overnight.

Gelation speed was studied by rheometry using an Anton Paar Physica MCR 301 device with a 25 mm rotating head diameter. Chitosan solutions and Na-β-GP and Na-β-GP/Mg-GP solution-suspensions were prepared as described above, pre-cooled to 4 °C, mixed at 4 °C and immediately subsequently subjected to a time sweep measurement at 37 °C at a strain of 0.01% and an angular frequency of 10 rad/s for 5000 seconds.

### 3.2. Mineralization Studies: Amount and Type of Mineral Formed

Hydrogel samples were removed from tubes and cut into discs 5 mm in thickness and incubated for 14 days in SBF with refreshment every 2 days. SBF was prepared using a protocol based on the method of Kokubo [37] with the addition of 10 mM Na-GP (as a substrate for ALP). After 14 days, hydrogels were rinsed three times with Milli-Q water, weighed, freeze dried for 48 h and reweighed. The dry mass percentage was calculated as: (weight after incubation and subsequent freeze-drying for 48 h/weight before freeze-drying) × 100. After mineralization studies and freeze-drying, the molecular structure of the hydrogels was investigated using Attenuated Total Reflectance Fourier-Transform Infrared (ATR-FTIR) spectroscopy as described previously [38]. Transmission Electron Microscopy (TEM) images were taken in bright field mode and Selected Area Electron Diffraction (SAED) patterns were obtained as described previously. Total concentrations of Ca were determined using flame atomic absorption spectrophotometry (AAS) after digestion. A fragment of each dried hydrogel type was weighed on a microbalance to the nearest 0.1 mg and was thermally digested at 550 °C for 5 h. Residues were dissolved in concentrated HNO_3_ (14 mol/L), and diluted 20 times to a final concentration of 5% HNO_3_ (v/v). The concentration of Ca was determined using flame AAS (SpectrAA100, Varian, Mulgrave, Australia), after calibration with 4 standards in the ranges 0 to 1.00 mg/L. Calibration was checked with an independent reference material, for which the maximum deviation is 7.5% (certificate). A procedural blank was analyzed and showed results lower than the quantification limits (0.1 µg/mg for a 10 mg sample). Concentrations of the samples were expressed as μg Ca per mg dry weight of the material.

### 3.3. Cell Culture Studies

Prior to cell culture studies, chitosan, Na-β-GP and Mg-GP were sterilized using ethylene oxide (EO) as described previously [30]. ALP solution was sterilized by filtration through a filter of pore diameter 0.22 μm. The hydrogels were prepared under sterile conditions. Discs of diameter 6 mm and height 2 mm were cut out of the formed hydrogels with a hole punch. Cells of the human osteoblastic cell line MG-63 were seeded onto hydrogel samples. Each hydrogel sample was placed in a well of a 24-well plate and a suspension of 100,000 cells in 1.5 mL cell culture medium, *i.e.* High Glucose DMEM containing 10% fetal bovine serum, 0.1 mM Sodium Pyruvate and 1% Penicilin-Streptomycin, (all Gibco, Invitrogen, Carlsbad, CA, USA), was added. To visualize cell attachment and distribution on the hydrogels, the cell cultures were evaluated using fluorescence microscopy.

A Live-Dead staining (Calcein AM/propidium iodide, Life Technologies, Eugene, OR, USA) was performed to evaluate cell viability. After rinsing, the supernatant was replaced by 1 mL PBS solution supplemented with 2 μL (1 mg/mL) calcein AM and 2 μL (1 mg/mL) propidium iodide. Cultures were incubated for 10 min at 37 °C, washed with PBS solution and evaluated by fluorescence microscopy (Type U-RFL-T, Olympus, Aartselaar, Belgium). Evaluations were performed 2 days and 4 days post-seeding. Eluates were produced by incubating four hydrogel samples in 2 ml of cell culture medium at 37 °C for 48 h. Eluates were diluted by factors of 1 (undiluted), 2, 4, 8, and 16. MG-63 cells (10,000 per well of a 96-well plate) were subsequently incubated in eluate at the aforementioned dilutions for 72 h. Vitality of the cells incubated in eluates was evaluated using the colorimetric MTT assay, using a 3-(4, 5-dimethyldiazol-2-yl)-2, 5-diphenyltetrazolium bromide (MTT, Cell Proliferation Kit I, Roche, Mannheim, Germany). The eluates were then replaced by 0.2 mL (0.5 mg/mL) MTT reagent diluted in cell culture medium. After 4 h incubation at 37 °C, the MTT reagent was removed and replaced by 0.2 mL lysis buffer (1% Triton X-100 in isopropanol/0.04 M HCl) for 30 min. A quantity of 0.2 mL of the resulting dissolved formazan solution was used for the spectrophotometric measurement at 580 nm (Versa Max Microplate Reader, Molecular Devices Corporation, Sunnyvale, CA, USA). Triplicate measurements were performed. The viability was calculated as a percentage of control cultures incubated with cell culture medium without eluate.

### 3.4. Statistical Analysis

Data were presented as mean ± S.D. (Standard Deviation). Multiple comparison procedures were performed with ANOVA. A value of *P* ≤ 0.05 was considered significant.

## 4. Conclusions

Chitosan hydrogels could be formed by partial substitution of Na-β-GP with Mg-GP at a molar ratio of 90:10. Gelation speeds were similar for chitosan/Na-β-GP and chitosan/Na-β-GP/Mg-GP hydrogels. Both hydrogel types were mineralized with apatite to a similar degree after incubation in SBF for 7 days. Both hydrogel types showed good and comparable cytocompatibility.
